# Intensive care-related loss of quality of life and autonomy at 6 months post-discharge: Does COVID-19 really make things worse?

**DOI:** 10.1186/s13054-022-03958-6

**Published:** 2022-04-04

**Authors:** Fabrice Thiolliere, Claire Falandry, Bernard Allaouchiche, Victor Geoffray, Laurent Bitker, Jean Reignier, Paul Abraham, Stephanie Malaquin, Baptiste Balança, Hélène Boyer, Philippe Seguin, Céline Guichon, Marie Simon, Arnaud Friggeri, Charles-Hervé Vacheron, AZUREA Study Group, Laurent Argaud, Bernard Floccard, Thomas Rimmele, Albrice Levrat, Stanislas Ledechowski, Remi Bruyere, Carole Schwebel, Benedicte Zerr, Luc Jarrige, Quentin Blanc, Jerome Morel, Olivier Baldesi, Gaëtan Plantefeve, Philippe Seguin, Claire Dahyot-Fizelier, Michel Bonnivard, J. Roustan, S. Vimeux, Ali Mofredj, Sami Alaya, Adel Maamar, Julio Badie, Bertrand Souweine, Gerald Choukroun, Oriane Fontaine, Jean Michel Constantin, Marc Gainier, Benoit Misset, Jean Claude Orban, Jean Reignier, Jean-Marc Doise, Olivier Millet, Laurent Favier, Berangere Jany, Ramin Ravan, Delphine Roux, Pierre Marie Bertrand, Nicolas Bele, Stéphanie Malaquin, Pierre Grégoire Guinot, Jean Pierre Quenot, Fanny Bounes, Claude Koubi, P. Danin

**Affiliations:** 1 Département d’Anesthésie Réanimation, Centre Hospitalier Lyon Sud, Hospices Civils de Lyon, Lyon, France; 2 Hospices Civils de Lyon, Geriatrics Unit, Centre Hospitalier Lyon Sud, Pierre-Bénite, France; 3 University of Lyon, CarMeN Laboratory, Inserm U1060, INRA U1397, Université Claude Bernard Lyon 1, INSA Lyon, Charles Mérieux Medical School, Pierre-Bénite, France; 4 Université Claude Bernard, Lyon1, Villeurbanne, France; 5 Université de Lyon, VetAgro Sup, Campus Vétérinaire de Lyon, UPSP 2016.A101, Pulmonary and Cardiovascular Aggression in Sepsis, 69280 Marcy l’Étoile, France; 6 Service de Médecine Intensive – Réanimation, Hôpital de La Croix Rousse, Hospices Civils de Lyon, Lyon, France; 7 Université Claude Bernard, Lyon 1, INSA-Lyon, UJM-Saint Etienne, CNRS, Inserm, CREATIS, UMR 5220, U1206, Université de Lyon, 69621 Lyon, France; 8 Service de Médecine Intensive Réanimation, CHU de Nantes, Nantes, France; 9 Service d’Anesthésie-Réanimation, Hôpital Édouard Herriot, 69008 Hospices civils de LyonLyon, France; 10 Réanimation chirurgicale, CHU amiens, Lyon, France; 11 Hospices Civils de Lyon, service d’anesthésie réanimation neurologique, Hôpital Pierre Wertheimer, département d’anesthésie reanimation, 59 Boulevard Pinel, 69500 Bron, France; 12 Centre de Recherche en Neurosciences de Lyon, U1028, Bron, France; 13 Direction de la Recherche en Santé, Hospices Civils de Lyon, Lyon, France; 14 Réanimation chirurgicale. CHU Rennes, 2 rue Henri Le Guilloux, 35000 Rennes, France; 15 Département d’anesthésie et réanimation chirurgicale, hôpital Croix Rousse, Hospices Civils de Lyon, Lyon, France; 16 Laboratoire Inter universitaire de Biologie de la Motricité (LIBM), Lyon, France; 17 Médecine Intensive - Réanimation- Hôpital Edouard Herriot, 1 place d’Arsonval, 69003 Lyon, France; 18 Département d’Anesthésie Réanimation, Centre Hospitalier Lyon Sud Hospices Civils de Lyon, Pierre-Bénite, France; 19 CIRI, Centre International de Recherche en Infectiologie (Equipe Laboratoire des Pathogènes Emergents), Inserm, U1111, Université Claude Bernard Lyon 1, CNRS, UMR5308, Lyon, France; 20 Faculté de Médecine Lyon Est, Université Claude Bernard Lyon 1, Lyon, France; 21 Pôle Santé Publique, Service de Biostatistique - Bioinformatique, 165, chemin du grand revoyet, 69495 Pierre-Bénite, Lyon, France

**Keywords:** COVID-19, Autonomy, Quality of life

## Abstract

**Objective:**

To compare old patients hospitalized in ICU for respiratory distress due to COVID-19 with old patients hospitalized in ICU for a non-COVID-19-related reason in terms of autonomy and quality of life.

**Design:**

Comparison of two prospective multi-centric studies.

**Setting:**

This study was based on two prospective multi-centric studies, the Senior-COVID-Rea cohort (COVID-19-diagnosed ICU-admitted patients aged over 60) and the FRAGIREA cohort (ICU-admitted patients aged over 70).

**Patients:**

We included herein the patients from both cohorts who had been evaluated at day 180 after admission (ADL score and quality of life).

**Interventions:**

None.

**Measurements and main results:**

A total of 93 COVID-19 patients and 185 control-ICU patients were included. Both groups were not balanced on age, body mass index, mechanical ventilation, length of ICU stay, and ADL and SAPS II scores. We modeled with ordered logistic regression the influence of COVID-19 on the quality of life and the ADL score. After adjustment on these factors, we observed COVID-19 patients were less likely to have a loss of usual activities (aOR [95% CI] 0.47 [0.23; 0.94]), a loss of mobility (aOR [95% CI] 0.30 [0.14; 0.63]), and a loss of ADL score (aOR [95% CI] 0.30 [0.14; 0.63]). On day 180, 52 (56%) COVID-19 patients presented signs of dyspnea, 37 (40%) still used analgesics, 17 (18%) used anxiolytics, and 14 (13%) used antidepressant.

**Conclusions:**

COVID-19-related ICU stay was not associated with a lower quality of life or lower autonomy compared to non-COVID-19-related ICU stay.

**Supplementary Information:**

The online version contains supplementary material available at 10.1186/s13054-022-03958-6.

## Introduction

The coronavirus disease 2019 (COVID-19) pandemic has been affecting the global population for the past year, has had a major impact on the number of hospital admissions including a high proportion of patients presenting acute respiratory failure, and has put a strain on the flow of patients admitted into intensive care units (ICUs). Indeed, approximatively 15% of hospitalized COVID-19 patients require ICU admission [1, 2].

For long, it has been known that the physical and psychological impact of an ICU stay on patients could be significant and prolonged. For ICU survivors, functional disability may persist for many years after hospital discharge, particularly in cases of Acute Respiratory Distress Syndrome (ARDS) [3]. This long-lasting disability depends on the intensity and duration of sedation and on the length of the ICU stay. Many studies have focused on the morbidity attributable to ICU care, including the physical, cognitive, psychological, and social consequences of hospitalization, as well as on the factors impacting the quality of life post-discharge [4–6]. Several studies have documented the long-term consequences of previous epidemic episodes of coronavirus infection (Severe Acute Respiratory Syndrome and Middle East Respiratory Syndrome) on functional and psychological impairments [7]. Such long-term symptomatology has also been identified in the COVID-19 context and has been named “long-COVID” [2, 8].

To date, little information has been published on the long-term sequelae of older patients who have survived a COVID-19-related ICU stay, and most studies have included small descriptive cohorts and focused on the day-180 status [9, 10], except for the recent study from Hodgson et al*.* that reported adequate data on the functional outcome at 6th months of 117 patients [11]. The question of the future quality of life of the large proportion of older COVID-19 ICU-admitted patients, given their initial medical conditions, deserves special attention [12–14].

The long-term consequences on the quality of life of the ICU stay and of long COVID are probably intertwined, and both might even have a synergic effect. The aim of the present study was to compare older patients hospitalized in ICU for respiratory distress due to COVID-19 with older patients hospitalized in ICU for a non-COVID-19-related reason, in terms of long-term autonomy and quality of life.

We therefore used two prospective cohort studies: the SENIOR-COVID study (a multicenter prospective cohort study carried out during the beginning wave of the pandemic) and the FRAGIREA study (carried out between 2018 and 2019, focusing on the long-term outcome of older patients managed in ICU).

## Materials and methods

### Study design

#### COVID-19 cohort

The COVID-19 cohort was built from the Senior-COVID-Rea study. The Senior-COVID-Rea study was a retrospective and prospective multicenter study on health data, carried out in 7 ICUs in the Auvergne-Rhône-Alpes region (France) for patients admitted between March 1, 2020 and May 6, 2020 during the first wave of the COVID-19 pandemic. The study protocol (V1.0 of April 7, 2020) was approved by the Ethics Committee of the *Hospices Civils de Lyon* on May 12, 2020 *(IRB number 20_025*) and declared on the ClinicalTrials platform on June 9, 2020 (NCT04422340). The detailed protocol was published elsewhere [15]. All patients over 60 years old, admitted to ICU in the participating centers during the study period with a COVID-19 diagnosis confirmed by positive SARS-CoV-2 PCR on nasopharyngeal or lung swabs were included in this cohort. In several centers, patients were contacted between days 173 and 187 after the day of ICU admission by a routine post-ICU teleconsultation (day 180 follow-up). In the centers routinely performing post-ICU teleconsultation, the data collected were incorporated in the Senior-COVID-Rea database.

#### Control cohort

The control cohort was built from the FRAGIREA study, which was a multicenter prevalence study on the frequency of frailty and the management of older patients [16]. The study was approved by the French data protection agency (*Commission nationale de l’informatique et des libertés*) and by an ethics committee (*Comité de Protection des Personnes Ouest II IRB Number 17.11.66*). The protocol was submitted to clinicaltrial.gov (NCT03326635). The study was conducted in 40 French ICUs, with the support of the AZUREA network (Additional file 1: Table S1).

Recruitment was conducted from April 2018 to January 2019. All patients included in the study were followed for 6 months or until death. The 6-month follow-up ended in July 2019. All patients aged 70 years or older, who were hospitalized in an ICU with an expected length of stay of more than 48 h, were eligible for inclusion. During this pre-pandemic study, a teleconsultation was conducted following the same protocol as the one described above for the COVID-19 cohort.

#### Patient exclusion

In both cohorts, patients who died before day 180, patients without a systematic consultation on day 180, or patients who were lost to follow-up, were excluded, and for the control cohort, patients admitted for a traumatic or surgical diagnosis were also excluded.

### Data collection

The data collected consisted in social and demographic data (age, sex, body mass index [BMI]), previous clinical autonomy (ADL score before admission), place of living, severity at admission (SAPS II), mechanical ventilation, renal replacement therapy, need of vasopressor, and length of ICU stay. For the day-180 consultation, autonomy (ADL score), quality of life regarding usual activities, anxiety, pain or discomfort, and mobility information were collected according to the EQ5D score (except autonomy assessed with the ADL score). For COVID-19 patients, data collected at day 180 included the IADL (instrumental activities of daily living) score, degree of dyspnea measured using the modified Medical Research Council (mMRC) dyspnea scale, number of medical consultations since discharge, consumption of anxiolytics, antidepressants, and analgesics. Autonomy was measured using 2 scales: a functional evaluation was performed using the ADL scale based on 6 activities of daily living, and a more refined evaluation was performed using the IADL scale, based on 8 more complex tasks using instruments of daily living (measured only for the COVID-19 patients) [17].

### Objective

We sought to assess the specific association of ICU admission related to COVID-19 on patient quality of life and autonomy (at day 180 post-ICU admission).

### Statistical analysis

Continuous variables were expressed as median (m) and [interquartile range, IQR], and categorical variables were expressed as count (percentage). Differences between groups were tested using the Wilcoxon rank sum test, chi-square test, or Fisher test.

For the modeling of the association of COVID-19-related ICU stay on the quality of life and autonomy, we used an ordinal logistic regression model on the different variables. Variables were ordered as categorical variable from the highest quality of life to the lowest quality of life. For the ADL score, the variable was ordered from the higher score (6, higher autonomy) to the lower score (0, lower autonomy). The ordered logistic regression allowed to estimate odds ratio (OR) and their associated 95% confidence interval (95% CI). Briefly, the OR derived from an ordinal logistic regression model represent the odds associated with the increase in one level in the ordered factor. As an illustrative example, the association of the COVID-19-related ICU stay on the usual activity (ordered as No problem; Some Problems; A lot of problems) will be, for an OR < 1, a “protective factor” of the increase in the variable (and protective factor of the ability to maintain usual activity), for an OR > 1, a promoting factor of the increase in the variable (and interfering with the ability to maintain usual activity), and for an OR = 1, a factor having no association on the usual activity. Finally, adjusted OR (aOR) were also estimated, they were adjusted on the main baseline characteristic that were not well balanced between both cohorts (p value < 0.05 in univariate analysis). These analyses were also performed on subgroups of patients aged over 70 years old as a sensitivity analysis.

P-values < 0.05 were considered as significant. Analyses were performed using R software version 3.6.4, and the package MASS.

## Results

**Fig. 1:**
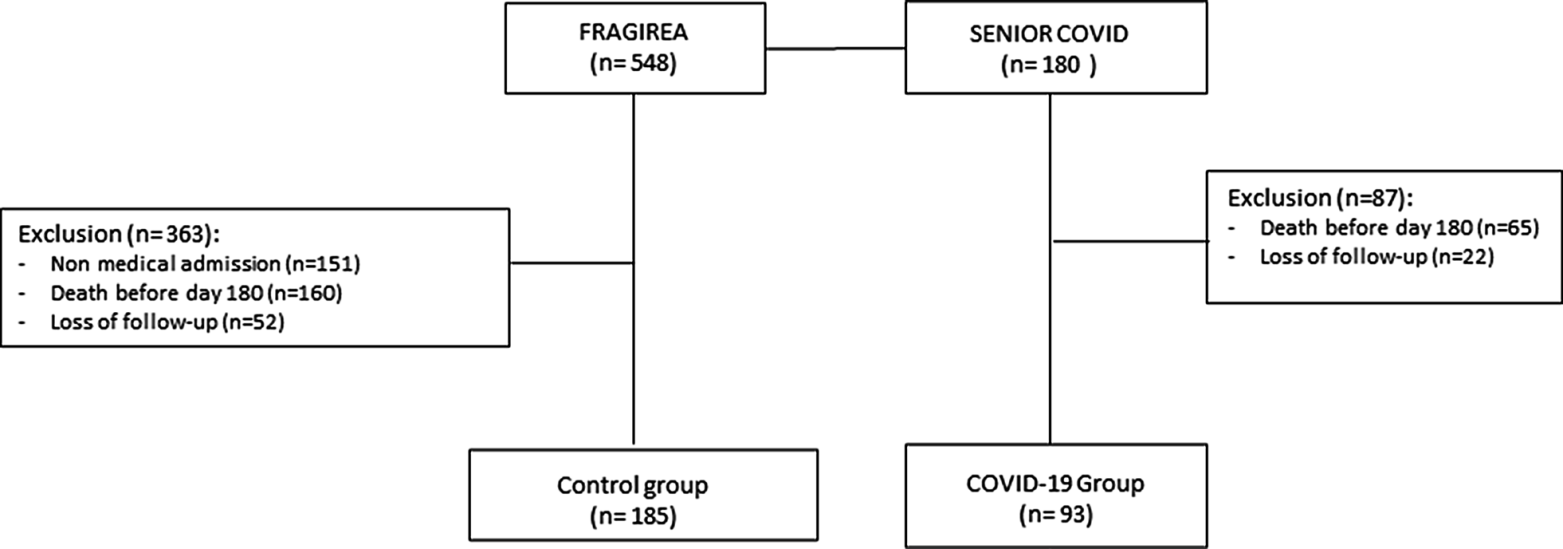
Flowchart

In the Senior-COVID-Rea study, 180 patients were included. At day 180, 65 (36.1%) patients had died, and 22 were lost to follow-up. Finally, 93 patients were included in the COVID-19 group of the present study (Fig. 1).

Among the 548 patients included in the FRAGIREA cohort, 160 (29.1%) patients had died at day 180, 52 were lost to follow-up, and 151 were excluded for non-medical admission as described in the methodology (characteristics of the patients excluded from the study available in Additional file 1: Table S2). Finally, 185 patients were included in the control group of the present study (Fig. 1).

Both groups were imbalanced in terms of baseline characteristics: patients in the COVID-19 group were younger (m [IQR]: 71 [65–76] years vs 78 [73–82] years), had a higher BMI, higher autonomy, and lower SAPS II. Also, the proportion of patients placed under mechanical ventilation or requiring vasopressor was lower among the COVID-19 group. However, the length of ICU stay was longer in the COVID-19 group compared to the control group (m [IQR]: 20 [7–40] days vs 7 [5–11] days; Table 1).

**Table 1:** Main baseline characteristics

Variables	Control group (n = 185)	COVID-19 group (n = 93)	*p* value
Age, years	78 [73–82]	71 [65–76]	< 0.001
Male sex	105 (56.8)	61 (65.6)	0.198
BMI, kg/m^2^	26 [23–30]	27 [25–30]	0.036
*Place of living*			0.113
Home	149 (80.5)	81 (88.0)	
Home with help	29 (15.7)	11 (12.0)	
Institution	7 (3.8)	0 (0)	
ADL score	6.0 [5.5–6.0]	6.0 [6.0–6.0]	< 0.001
SAPS II	49 [39–59]	38 [31–45]	< 0.001
Mechanical ventilation	89 (48.1)	60 (64.5)	0.014
Renal replacement therapy	17 (9.2)	7 (7.5)	0.811
Vasopressor	105 (56.8)	28 (30.1)	< 0.001
Length of stay in ICU, days	7 [5–11]	20 [7–40]	< 0.001

Results are expressed as count (percentage) or median [interquartile range, IQR]. p values for the comparison between groups

BMI, body mass index; ADL, activities of daily living; SAPS, simplified acute physiology score; ICU, intensive care unit

**Fig. 2:**
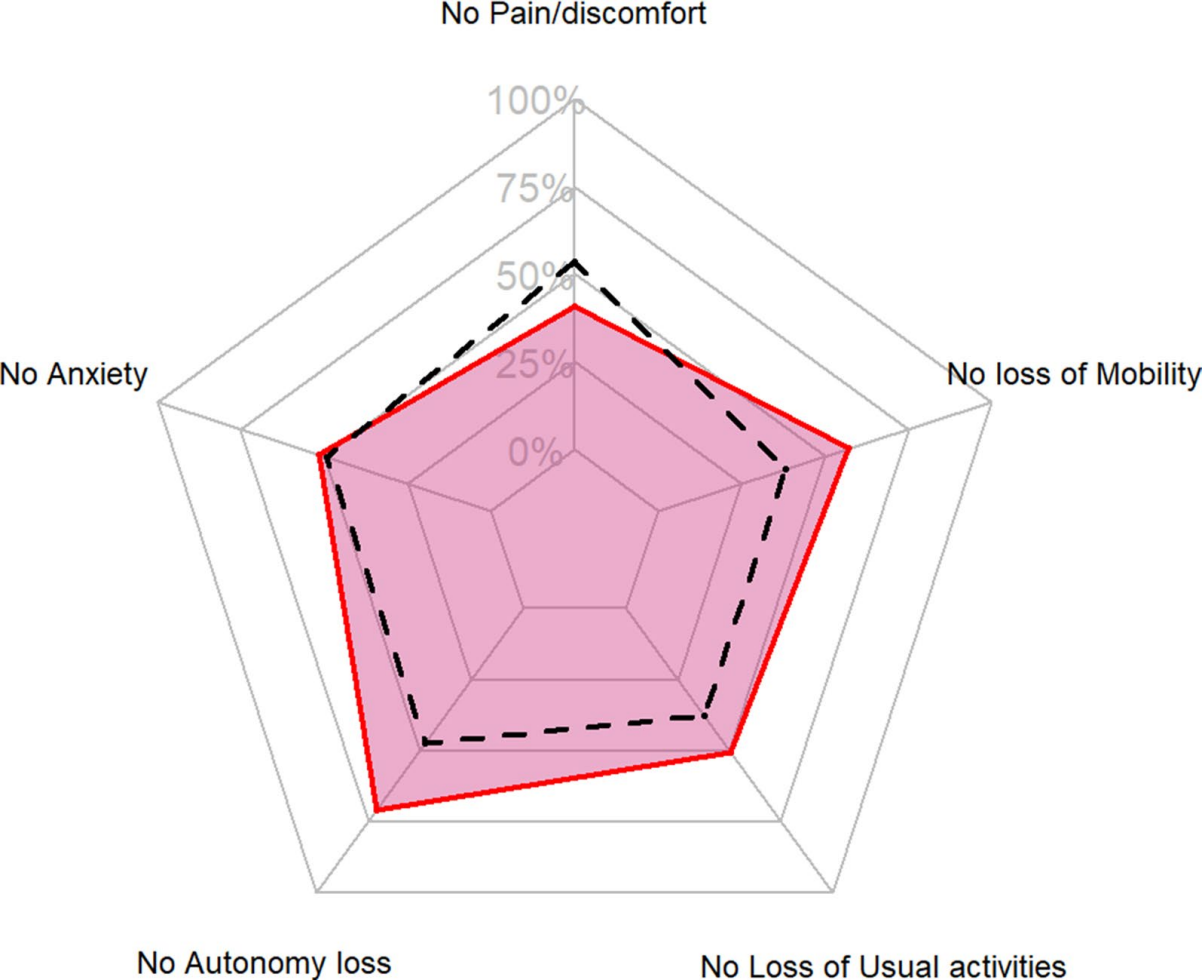
Spider chart of the loss of quality of life and autonomy at day 180. Proportion of patients with no pain or discomfort; no loss of mobility; no loss of usual activities; no loss of autonomy (ADL score at 6); and no anxiety. Red line: COVID-19 patients; Black dotted line: control group

Regarding the different dimensions of the EQ5D score at day 180, and after adjustment, COVID-19 patients were less likely to have problems in their usual activities (aOR [95% CI] 0.47 [0.23; 0.94]) and had fewer mobility problems (aOR [95% CI] 0.30 [0.14; 0.63]). They also had a lower risk of loss of autonomy (based on the ADL score; aOR [95% CI] 0.30 [0.14; 0.63]). No difference was observed after adjustment for pain, anxiety, and autonomy (Table 2; Fig. 2). The results of the sensibility analysis (i.e., including only patients over 70 years old) were similar (Additional file 1: Table S3).

**Table 2:** Comparison of quality of life and autonomy on day 180 between COVID-19 and control groups

Variables	Control group (n = 185)	COVID-19 group (n = 93)	OR [95% CI]	aOR [95% CI]
*Usual activities*			0.58 [0.36; 0.93]	0.47 [0.23; 0.94]
No problem	70 (37.8)	47 (50.5)	*p* = 0.0235	*p* = 0.0361
Some problems	80 (43.2)	36 (38.7)		
A lot of problems	35 (18.9)	10 (10.8)		
*Anxiety*				
Not unhappy, sad, or worried	91 (49.2)	48 (51.6)	0.88 [0.54; 1.41]	0.67 [0.33; 1.36]
A bit unhappy, sad, or worried	73 (39.5)	37 (39.8)	*p* = 0.5929	*p* = 0.2727
Very unhappy, sad, or worried	21 (11.4)	8 (8.6)		
*Pain/discomfort*			0.9 [0.57; 1.42]	0.89 [0.46; 1.70]
No pain/discomfort	99 (53.5)	38 (40.9)	*p* = 0.6600	*p* = 0.7228
Some pain/discomfort	18 (9.7)	43 (46.2)		
A lot of pain/discomfort	68 (36.8)	12 (12.9)		
*Mobility*			0.47 [0.28; 0.77]	0.30 [0.14; 0.63]
No problem	71 (38.4)	53 (57)	*p* = 0.003	*p* = 0.0016
Some problems	102 (55.1)	37 (39.8)		
A lot of problems	12 (6.5)	3 (3.2)		
ADL	5.5 [4.0–6.0]	6 [5.5–6.0]	0.35 [0.21; 0.58]	0.30 [0.14; 0.63]
			*p* = < 0.0001	*p* = 0.0019

Results are expressed as count (percentage) or median [interquartile range]. Adjustment on the age, BMI, ADL score, SAPS II, length of ICU stay, mechanical ventilation during ICU stay, and vasopressor requirement. For the ADL score, the variable was ordered from the higher score (6, higher autonomy) to the lower score (0, lower autonomy)

OR, Odds ratio; aOR, adjusted odds ratio, 95% CI, 95% confidence interval; ADL, activities of daily living; BMI, body mass index; SAPS, simplified acute physiology score; ICU, intensive care unit

At day 180, 52/93 (56%) COVID-19 patients still suffered from dyspnea, and 37 (40%) used analgesics for pain. A high proportion of them also consumed anxiolytic (17, 18%) and antidepressants (13, 14%). Their median [IQR] dependence score (IADL score) was 8 [4–8] (Table 3). Finally, 59 (63%) COVID-19 patients evaluated at 180 days had an IADL score > 5 and 49 (53%) had an IADL score = 8.

**Table 3:** Characteristic of the COVID-19 patients at day 180 post-ICU admission

Variables	COVID-19 patients (n = 93)
IADL score	8 [4–8]
mMRC score	1 [0–2]
Presence of dyspnea (mMRC > 0)	52 (56.5)
Presence of severe dyspnea (mMRC ≥ 2)	25 (26.8)
*Number of medical consultations since discharge*	
< 5	66 (71.7)
5–10	7 (7.6)
> 10	19 (20.7)
Anxiolytic consumption	17 (18.5)
Antidepressant consumption	13 (14.1)
*Analgesic consumption*	
Non-opioid analgesics	37 (40.2)
Weak opioids	17 (18.5)
Strong opioids	2 (2.2)

Results are expressed as count (percentage) or median [interquartile range]

IADL, instrumental activities of daily living; mMRC, modified medical research council

## Discussion

In the present study, we examined the long-term consequences of COVID-19-related ICU stay. Surprisingly, we showed that the impact of the ICU stay on the long-term outcome was not worse in case of admission for COVID-19-related reason compared to any other medical reason. The consequences on the quality of life and autonomy were nonetheless severe, as for any ICU stay.

Altogether, our results suggest that the impact of ICU stay on the long-term outcome of older survivors was similar and not worse in case of COVID-19-related reason compared to any other medical reason for ICU admission and corresponds to regular PICS. We confirmed this by using a control group from a previous cohort study, with similar inclusion criteria. We chose to target older patients because this population is the most likely to suffer from severe loss of quality of life or autonomy after an ICU stay. A recent study by Hodgson et al*.* has been recently published, and the design was similar to ours [11]. A total of 212 critically-ill ICU-admitted COVID-19 patients were included in that study, and the follow-up was adequate for 160 of them. The mortality was lower (26.9%) compared to the one we observed (36%), mainly because there was no restriction in terms of age in that cohort (median age at 62 years old), which also explains the shorter length of ICU stay [11].

Although that cohort was younger, our results are consistent with their study in terms of mobility issues, usual activities, pain/discomfort, and anxiety/depression. In our study, the prognosis at 6 months was a little worse, and overall 60% of patients presented a disability at 6 months [11]. These results are also consistent with the conclusions from a systematic review including 12 studies [14]. In their study, Hodgson et al*.* did not provide any comparison between COVID-19 and regular ICU patients [11], which is the major information provided by the present study.

Indeed, COVID-19 patients were more likely to maintain usual activities, or to have no mobility problem. Our descriptive results were similar to those of Gautam et al. who have analyzed the quality of life of 200 patients with severe COVID-19 [18]. Indeed, a similar proportion of patients had reduced mobility (about 4 in 10 in both studies). However, in the present study, there were more patients experiencing pain among COVID-19 patients compared to the control group, although the difference did not remain after adjustment. Long-COVID-19-related pain includes non-specific discomforts such as sore throat, body ache, headache, and myalgia. McCue et al*.* have reported that 67% of the long-COVID-19 patients had chronic pain, and for 29% of them, pain could be considered as severe[19]. Pain lasts long after the infection is cleared and may be the consequence of a deregulated host immune response to the infection[20]. In the present study, almost half of the COVID-19 ICU-admitted patients had chronic pain, and half of them required opioids.

The psychological impact of COVID-19 is not well known. About a third of severe COVID-19 patients have been showed to display signs of post-traumatic stress disorder[21]. However, in the general population of ICU survivors, 25% will suffer from PTSD[22]. This observation raises the question of how to distinguish Post Intensive Care Syndrome (PICS) from long-COVID. A study has even suggested there was a higher suicide risk during post-COVID-19 syndrome[23]. In the present study, half of the COVID-19 patients had anxiety on day 180, a fifth were taking anxiolytics, and 14% antidepressants. The prevalence of anxiety was not higher among the COVID-19 patients compared to the control group, but these results underline the importance of prevention and post-intensive care follow-up with adequate nursing care, environmental management, and psychological therapy[24]. These consequences might be prevented by an adequate psychological support[25].

We also found a large number of patients presenting dyspnea at 6-month post ICU admission, higher than the persistent dyspnea expected during the long-COVID. For example, Meije et al. have found that 10% of patients suffering from long-COVID were expected to have persistent dyspnea[10]. This difference might be explained by the fact that hospitalized patients were included in the latter study, and only 9% were ICU patients. Therefore, the difference might be related to the severity of COVID-19 or solely to the PICS among ARDS patients.

We should notice that these patient losses to follow-up were most likely not random, but the proportion of patient’s loss to follow-up was similar between both groups and expected when considering a 6-month outcome. Therefore, this parameter is unlikely to have induced a serious bias. The two main differences between both cohorts were their period of recruitment and their age at ICU admission. Indeed, the COVID-19 patients were younger, and we performed an adjustment of the Odds ratio on age to control this bias. Moreover, we performed a sensitivity analysis on the patients aged over 70 years, and we obtained similar results. While acknowledging its limitation, we used an univariate selection algorithm for the selection of confounders because of its wide use in intensive care studies and its easy understandability, and because of its easy implementation with an ordinal outcome[26]. Apart from the ADL score, the baseline quality-of-life characteristics were not collected in our study, and we could not adjust our results on these variables. We also did not assess the cognition of patients: as SARS-CoV-2 is a neurotropic virus, the cognition of COVID-19 patients could be worsened, this hypothesis needs to be explored in future study. Finally, we should also mention that the cohort of COVID-19 patients was built before the RECOVERY trial results were published: it has since then come to our attention that the use of steroids, particularly with neuromuscular blocking agent, increases the risk of long-lasting myopathy, and can alter the quality of life. It is difficult to distinguish the sole long-COVID from the PICS [27]. Indeed, each process occurs during the recovery period after the ICU stay is related to similar clinical presentations, and is probably intertwined. However, this emphasizes the importance of long-term follow-up of COVID-19 and non-COVID-19 ICU-admitted patients in order to detect and treat PICS with multidisciplinary therapy (physical, nutritional, and psychological) [28, 29]. However, even with a follow-up, ICU survivors have been reported to think that their healthcare needs are not adequately fulfilled after their ICU discharge[30].

## Conclusion

COVID-19 patients surviving an ICU stay do not have a worse outcome than regular ICU medical patients in the long term, but further studies including larger cohorts of patients followed-up for a longer duration are required to confirm these results. The consequences on the quality of life and autonomy are nonetheless severe, as for any ICU stay.

## Supplementary Information

**Additional file 1: Table S1.** AZUREA Study group: Inclusion center. **Table S2.** Characteristics of the patients not included in the study (Medical patients only). **Table S3.** Comparison of quality of life and autonomy on day 180 between COVID-19 and control groups restricted to the patients over 70 years old.

## Data Availability

Not applicable.
