# Supplementary material for: Intensive care-related loss of quality of life and autonomy at 6 months post-discharge: Does COVID-19 really make things worse?
Source: Crit Care. 2022 Apr 4;26:94. doi: 10.1186/s13054-022-03958-6 (PMC8978758; doi:10.1186/s13054-022-03958-6)
Supplement: Supplementary file 1 — Additional file 1: Table S1. AZUREA Study group: Inclusion center. Table S2. Characteristics of the patients not included in the study (Medical patients only). Table S3. Comparison of quality of life and autonomy on day 180 between COVID-19 and control groups restricted to the patients over 70 years old. [file 13054_2022_3958_MOESM1_ESM.docx]

**Supplementary table 1: AZUREA Inclusion Center**

| Name | First Name | City | Hospital | MAIL |
| --- | --- | --- | --- | --- |
| ARGAUD | LAURENT | Lyon | HCL, HEH, N REA | [laurent.argaud@chu-lyon.fr](mailto:laurent.argaud@chu-lyon.fr) |
| FLOCCARD | BERNARD | Lyon | HCL, HEH, G REA | [thomas.rimmele@chu-lyon,fr](mailto:thomas.rimmele@chu-lyon,fr) |
| RIMMELE | THOMAS | Lyon | HCL, HEH, P REA | [thomas.rimmele@chu-lyon,fr](mailto:thomas.rimmele@chu-lyon,fr) |
| LEVRAT | ALBRICE | Annecy | CH ANNECY | [alevrat@ch-annecygenevois.fr](mailto:alevrat@ch-annecygenevois.fr) |
| LEDECHOWSKI | STANISLAS | Bourgoin | CH BOURGOIN | [s.ledochowski@gmail.com](mailto:s.ledochowski@gmail.com) |
| BRUYERE | REMI | Bourg en Bresse | CH BOURG EN BRESSE | [rbruyere@ch-bourg01.fr](mailto:rbruyere@ch-bourg01.fr) |
| SCHWEBEL | CAROLE | Grenoble | CHU GRENOBLE Réa Med | [cschwebel@chu-grenoble.fr](mailto:cschwebel@chu-grenoble.fr) |
| ZERR | Benedicte | Chambéry | CH CHAMBERY | [jean.marc.thouret@ch-metropole-savoie.fr](mailto:jean.marc.thouret@ch-metropole-savoie.fr) |
| JARRIGE | LUC | Moulins | CH MOULINS | [l.jarrige@ch-moulins-yzeure.fr](mailto:l.jarrige@ch-moulins-yzeure.fr) |
| BLANC | QUENTIN | Valence | CH VALENCE | [qblanc@ch-valence.fr](mailto:qblanc@ch-valence.fr) |
| MOREL | JEROME | Saint Etienne | CHU SAINT ETIENNE Réa Poly | [jerome.morel@chu-st-etienne.fr](mailto:jerome.morel@chu-st-etienne.fr) |
| BALDESI | OLIVIER | Aix en Provence | CH AIX EN PROVENCE | [obaldesi@ch-aix.fr](mailto:obaldesi@ch-aix.fr) |
| PLANTEFEVE | GAËTAN | Argenteuil | CH ARGENTEUIL | [gaetan.plantefeve@ch-argenteuil.fr](mailto:gaetan.plantefeve@ch-argenteuil.fr) |
| SEGUIN | PHILIPPE | Rennes | CHU RENNES Réa Chir | [philippe.seguin@chu-rennes.fr](mailto:philippe.seguin@chu-rennes.fr) |
| DAHYOT-FIZELIER | CLAIRE | Poitiers | CHU POITIERS Réa Chir | [claire.dahyot-fizelier@chu-poitiers.fr](mailto:claire.dahyot-fizelier@chu-poitiers.fr) |
| BONNIVARD | MICHEL | Montauban | CH MONTAUBAN | [m.bonnivard@ch-montauban.fr](mailto:m.bonnivard@ch-montauban.fr) |
| ROUSTAN | J | Montauban | CH MONTAUBAN | [j.roustan@ch-montauban.fr](mailto:j.roustan@ch-montauban.fr) |
| VIMEUX | S | Montauban | CH MONTAUBAN | [s.vimeux@ch-montauban.fr](mailto:s.vimeux@ch-montauban.fr) |
| MOFREDJ | ALI | Salons de Provence | CH SALONS DE PROVENCE | [ali.mofredj@ch-salon.fr](mailto:ali.mofredj@ch-salon.fr) |
| ALAYA | Sami | Salons de Provence | CH SALONS DE PROVENCE | [sami.alaya@ch-salon.fr](mailto:sami.alaya@ch-salon.fr) |
| MAAMAR | Adel | Rennes | CHU RENNES Réa Med | [adel.maamar@chu-rennes.fr](mailto:adel.maamar@chu-rennes.fr) |
| BADIE | JULIO | Belfort | CH BELFORT | [Julio.BADIE@hnfc.fr](mailto:Julio.BADIE@hnfc.fr) |
| SOUWEINE | BERTRAND | Clermont Ferrand | CHU CLERMONT FERRAND Réa Med | [bsouweine@chu-clermontferrand.fr](mailto:bsouweine@chu-clermontferrand.fr) |
| CHOUKROUN | GERALD | Evry | CH SUD FRANCILIEN | [guillaume.chevrel@chsf.fr](mailto:guillaume.chevrel@chsf.fr) |
| FONTAINE | ORIANE | Mayotte | CH MAYOTTE | [o.fontaine@chmayotte.fr](mailto:o.fontaine@chmayotte.fr) |
| CONSTANTIN | JEAN MICHEL | Clermont Ferrand | CHU CLERMONT FERRAND Réa Chir | [efutier@chu-clermontferrand.fr](mailto:efutier@chu-clermontferrand.fr) |
| GAINIER | MARC | Marseille | AP-HM LA TIMONE Réa Med | [marc.gainnier@ap-hm.fr](mailto:marc.gainnier@ap-hm.fr) |
| MISSET | BENOIT | Rouen | CHU ROUEN Réa Med | [Fabienne.Tamion@chu-rouen.fr](mailto:Fabienne.Tamion@chu-rouen.fr) |
| ORBAN | JEAN CLAUDE | Nice | CHU NICE Pasteur 2 RMC | [orban.jc@chu-nice.fr](mailto:orban.jc@chu-nice.fr) |
| REIGNIER | JEAN | Nantes | CHU NANTES Réa Med | [Jean.Reignier@univ-nantes.fr](mailto:Jean.Reignier@univ-nantes.fr) |
| DOISE | JEAN-MARC | Chalon sur Saône | CH CHALONS SUR SAONE | [jean-marc.doise@ch-chalon71.fr](mailto:jean-marc.doise@ch-chalon71.fr) |
| MILLET | OLIVIER | Montélimar | CH MONTELIMAR | [olivier.millet@ch-montelimar.fr](mailto:olivier.millet@ch-montelimar.fr) |
| FAVIER | LAURENT | Bézier | CH BEZIERS | [laurent.favier@ch-beziers.fr](mailto:laurent.favier@ch-beziers.fr) |
| JANY | Berangere | Bézier | CH BEZIERS | [berangere.jany@ch-beziers.fr](mailto:berangere.jany@ch-beziers.fr) |
| RAVAN | RAMIN | Vichy | CH VICHY | [ramin.ravan@ch-vichy.fr](mailto:ramin.ravan@ch-vichy.fr) |
| ROUX | Delphine | Vichy | CH VICHY | [delphine.roux@ch-vichy.fr](mailto:delphine.roux@ch-vichy.fr) |
| BERTRAND | Pierre Marie | Cannes | CH CANNES Rea Poly | [N.CLEMENT@ch-cannes.fr](mailto:N.CLEMENT@ch-cannes.fr) |
| BELE | Nicolas | Fréjus | CH FREJUS | [nicolasbele@free.fr](mailto:nicolasbele@free.fr) |
| MALAQUIN | Stéphanie | Amiens | Réa Chir | [malaquin.stephanie@chu-amiens.fr](mailto:malaquin.stephanie@chu-amiens.fr) |
| GUINOT | Pierre Grégoire | Dijon | Réa Chir | [guinotpierregregoire@gmail.com](mailto:guinotpierregregoire@gmail.com) |
| QUENOT | Jean Pierre | Dijon | CHU DIJON Rea Med | [jean-pierre.quenot@chu-dijon.fr](mailto:jean-pierre.quenot@chu-dijon.fr) |
| BOUNES | Fanny | Toulouse RANGUEIL | CHU TOULOUSE Rea Poly | [bounes.f@chu-toulouse.fr](mailto:bounes.f@chu-toulouse.fr) |
| KOUBI | Claude | Nice | HPGS NICE | [ckoubi@hpgs,fr](mailto:ckoubi@hpgs,fr) |
| DANIN | P | Nice | HPGS NICE | [pdanin@hpgs,fr](mailto:pdanin@hpgs,fr) |

**Supplementary Table 2: Characteristics of the patients not included in the study (Medical patients only)**

1. **Including the dead and lost to follow-up patients**

| Variables | Control Group (n=212) | COVID-19 Group  (n=87) | p value |
| --- | --- | --- | --- |
| Age, years | 78 [74-82] | 76 [69-82] | 0.007 |
| Male sex | 139 (65) | 72 (82) | 0.005 |
| BMI, kg/m² | 25 [22-30] | 27 [22-29] | 0.393 |
| Place of living |  |  | 0.790 |
| Home | 154 (73) | 60 (71) |  |
| Home with help | 45 (21) | 17 (20) |  |
| Institution | 13 (6) | 7 (8) |  |
| ADL score | 6.0 [4.5-6.0] | 6.0 [6.0-6.0] | <0.001 |
| SAPS II | 54 [44-67] | 45 [37-53] | <0.001 |
| Mechanical ventilation | 111 (52) | 57 (65) | 0.051 |
| Renal replacement therapy | 41 (19) | 9 (10) | 0.085 |
| Vasopressor | 139 (66) | 35 (40) | <0.001 |
| Length of stay in ICU, days | 8 [5-13] | 9 [4-28] | 0.302 |

**b. Restricted to the patients who died**

| Variables | Control Group (n=160) | COVID-19 Group  (n=65) | p value |
| --- | --- | --- | --- |
| Age, years | 78 [74-82] | 78 [69-82] | 0.178 |
| Male sex | 103 (64) | 53 (81) | 0.018 |
| BMI, kg/m² | 25 [22-29] | 27 [23-30] | 0.050 |
| Place of living |  |  | 0.802 |
| Home | 111 (69) | 42 (67) |  |
| Home with help | 38 (24) | 15 (24) |  |
| Institution | 11 (7) | 6 (9) |  |
| ADL score | 6.0 [4.5-6.0] | 6.0 [5.5-6.0] | 0.006 |
| SAPS II | 58 [48-73] | 47 [39-54] | <0.001 |
| Mechanical ventilation | 96 (60) | 48 (74) | 0.071 |
| Renal replacement therapy | 35 (22) | 8 (12) | 0.142 |
| Vasopressor | 115 (72) | 33 (51) | 0.004 |
| Length of stay in ICU, days | 9 [6-14] | 10 [4-29] | 0.421 |

Results are expressed as count (percentage) or median [interquartile range].

Abbreviations: ADL, activities of daily living; BMI, body mass index; SAPS, simplified acute physiology score; ICU, intensive care unit.

**Supplementary Table 3: Comparison of quality of life and autonomy on day 180 between COVID-19 and control groups restricted to the patients over 70 years old**

| Variables | Control group (n=185) | COVID-19 group (+70yo)  (n=53) | OR [95%CI] | aOR [95%CI] |
| --- | --- | --- | --- | --- |
| Usual activities |  | | 0.59 [0.33; 1.05] | 0.53 [0.24; 1.13] |
| *No Problem* | 70 (37.8) | 26 (49.1) | *p=*0.0737 | *p=*0.0361 |
| *Some Problems* | 80 (43.2) | 22 (41.5) |  |  |
| *A lot of problems* | 35 (18.9) | 5 (9.4) |  |  |
| Anxiety |  |  |  |  |
| *Not unhappy, sad, or worried* | 91 (49.2) | 27 (50.9) | 0.94 [0.52; 1.69] | 0.79 [0.36; 1.69] |
| *A bit unhappy, sad, or worried* | 73 (39.5) | 20 (37.7) | *p=* 0.8466 | *p=* 0.2727 |
| *Very unhappy, sad, or worried* | 21 (11.4) | 6 (11.3) |  |  |
| Pain/discomfort |  | | 0.80 [0.46; 1.38] | 0.96 [0.46; 1.98] |
| *No pain/discomfort* | 99 (53.5) | 25 (47.2) | *p=* 0.4252 | *p=* 0.7228 |
| *Some pain/discomfort* | 18 (9.7) | 21 (39.6) |  |  |
| *A lot of pain/discomfort* | 68 (36.8) | 7 (13.2) |  |  |
| Mobility |  |  | 0.54 [0.29; 0.99] | 0.36 [0.15; 0.81] |
| *No problem* | 71 (38.4) | 29 (54.7) | *p=* 0.0474 | *p=* 0.0162 |
| *Some problems* | 102 (55.1) | 21 (39.6) |  |  |
| *A lot of problems* | 12 (6.5) | 3 (5.7) |  |  |
| ADL | 5.5 [4.0-6.0] | 6 [5.0-6.0] | 0.5 [0.27; 0.89] | 0.32 [0.14; 0.72] |
|  |  |  | *p=* 0.0216 | *p=* 0.0066 |

Results are expressed as count (percentage) or median [interquartile range]. Adjustment on the age, BMI, ADL score, SAPS II, length of ICU stay, mechanical ventilation during ICU stay, and vasopressor requirement.

For the ADL score, the variable was ordered from the higher score (6, higher autonomy) to the lower score (0, lower autonomy).

Abbreviations: OR, Odds ratio; aOR, adjusted odds ratio, 95%CI, 95% confidence interval; ADL, activities of daily living; BMI, body mass index; SAPS, simplified acute physiology score; ICU, intensive care unit.
